# 3PFDB - A database of Best Representative PSSM Profiles (BRPs) of Protein Families generated using a novel data mining approach

**DOI:** 10.1186/1756-0381-2-8

**Published:** 2009-12-04

**Authors:** Khader Shameer, Paramasivam Nagarajan, Kumar Gaurav, Ramanathan Sowdhamini

**Affiliations:** 1 National Centre for Biological Sciences, Tata Institute of Fundamental Research, GKVK Campus, Bellary road, Bangalore 560065, India; 2 Department of Chemistry and Biomolecular sciences, Macquarie University, Sydney NSW, Australia

## Abstract

**Background:**

Protein families could be related to each other at broad levels that group them as superfamilies. These relationships are harder to detect at the sequence level due to high evolutionary divergence. Sequence searches are strongly directed and influenced by the best representatives of families that are viewed as starting points. PSSMs are useful approximations and mathematical representations of protein alignments, with wide array of applications in bioinformatics approaches like remote homology detection, protein family analysis, detection of new members and evolutionary modelling. Computational intensive searches have been performed using the neural network based sensitive sequence search method called FASSM to identify the Best Representative PSSMs for families reported in Pfam database version 22.

**Results:**

We designed a novel data mining approach for the assessment of individual sequences from a protein family to identify a single Best Representative PSSM profile (BRP) per protein family. Using the approach, a database of protein family-specific best representative PSSM profiles called 3PFDB has been developed. PSSM profiles in 3PFDB are curated using performance of individual sequence as a reference in a rigorous scoring and coverage analysis approach using FASSM. We have assessed the suitability of 10, 85,588 sequences derived from seed or full alignments reported in Pfam database (Version 22). Coverage analysis using FASSM method is used as the filtering step to identify the best representative sequence, starting from full length or domain sequences to generate the final profile for a given family. 3PFDB is a collection of best representative PSSM profiles of 8,524 protein families from Pfam database.

**Conclusion:**

Availability of an approach to identify BRPs and a curated database of best representative PSI-BLAST derived PSSMs for 91.4% of current Pfam family will be a useful resource for the community to perform detailed and specific analysis using family-specific, best-representative PSSM profiles. 3PFDB can be accessed using the URL: http://caps.ncbs.res.in/3pfdb

## Background

Sensitive sequence search techniques play a vital role in enhanced function annotation approaches for several gene products in the post genomic era. The deluge of sequence data generated by high-through put experiments need to be rapidly and effectively annotated using sensitive sequence search methods to understand the biological implications of individual sequences. Due to the practical inability of biochemical validation of large number of individual sequences from genome projects, bioinformatics tools are extensively developed and applied to enhance the function annotation of sequence and structural data [1-5]. BLAST [5] suite of programs are the first choice for such annotation of individual protein sequences based on homology and sequence conservation parameters. Position Specific Iterative BLAST (PSI- BLAST) [5] is one of the best variants among the BLAST programs that offer a sensitive sequence search method for searching the homologous sequences and representing the amino acid conservation at different alignment positions into mathematical patterns using Position Specific Scoring Matrices (PSSM).

PSSM [6-8] is a useful approximation of sequence alignments that can be easily integrated in to a variety of tools and bioinformatics software packages designed for specific applications [9,10]. PSI-BLAST-generated position-specific scoring matrices can be used in domains of bioinformatics like pattern recognition, machine learning, database searches, remote homology detection, prediction of transcription factors etc. In this paper, we report a novel data mining method that could be used to select a Best Representative PSSM profile (BRP) from a set of sequence of a protein family and the availability of a database of BRPs built on Pfam alignments subsequent to extensive analysis of individual members in a sequence family using FASSM (Function Association using Sequence & Structure Motifs) method [9].

FASSM examines the sequence conservation and positions of protein family signatures or motifs for the annotation of protein sequences and to facilitate the analysis of their domains. Residues that characterize motifs at different alignment positions can be identified using PSIMOT option in FASSM algorithm. FASSM method is driven by a neural network routine and was shown to be useful for difficult relationships such as discontinuous domains during whole-genome surveys and is demonstrated to perform accurate family associations at sequence identities as low as 15% [9]. In the present instance, FASSM algorithm and coverage analysis based on FASSM scoring is used to assess the ability of a sequence in a given protein family to generate the best-representative PSSM profiles. A database of "Best Representative PSSM profiles" (BRPs) of protein families (3PFDB) [11] is developed using a computationally intensive data-curation protocol that assessed 1.08 million PSI-BLAST generated PSSMs to identify the BRPs for 8,524 Pfam families. We also propose strategies for dealing with Pfam families where the associations of BRPs were not straightforward. The method that we have designed to obtain BRPs can be applied in general using any other program that requires PSSMs to obtain the BRPs. We envisage that the method will be useful for the community to derive BRPs from specific data sets along with the database.

## Construction and content

### Data Curation

**Figure 1:**
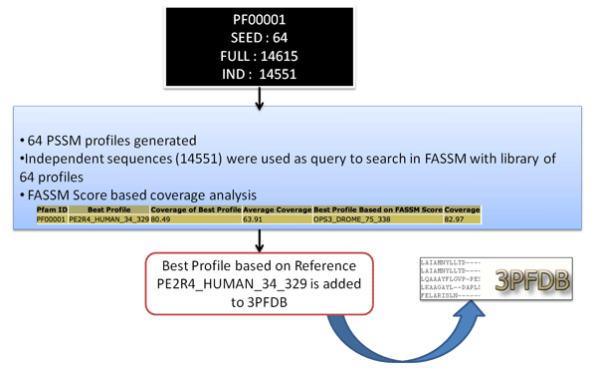
**Simplified graphical representation of the data curation to identify BRP of Pfam family PF00001**.

**Figure 2:**
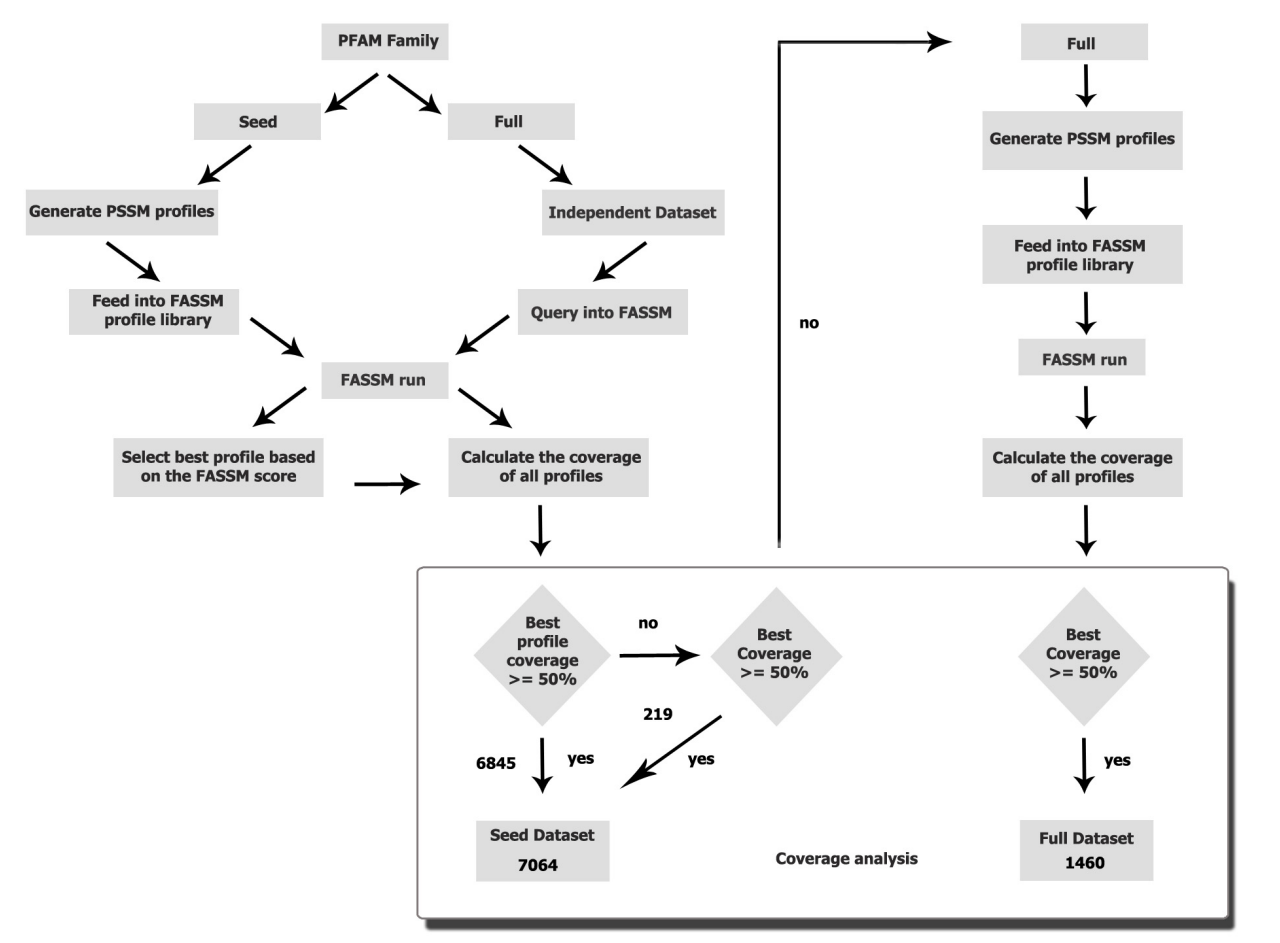
**Detailed flow chart of the data curation steps in 3PFDB**.

Family specific, Best Representative PSSM profiles in 3PFDB are identified using a computationally intensive exploratory search protocol. Every sequence in the (seed or full) alignment of a given protein family is given a chance to be the reference sequence and coverage analysis is performed using individual FASSM runs. Simplified graphical representation of the approach used to curate BRP of Pfam family PF00001 is provided in Figure 1. Different approaches based on seed and full datasets are followed to assess the suitability of a profile to be included in 3PFDB as the best-representative of a given family. We have used Pfam version 22 (October 2007) [12] for the data curation and 3PFDB database development. In this search protocol, we have successfully identified BRPs of 91.4% of Pfam families in release Pfam 22. Detailed flow chart of the data curation steps is provided in Figure 2.

**Figure 3:**
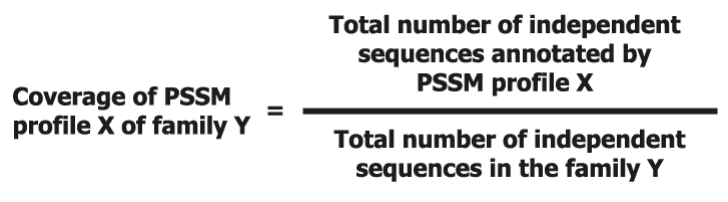
**3PFDB - Coverage analysis formula**.

Steps in 3PFDB data curation:

1. For a given Pfam family, PSI-BLAST derived PSSM profiles were generated for sequences in 'Seed sequence dataset' using PSI-BLAST search against the sequences in seed alignment.

2. Profiles generated in Step 1 are fed into FASSM profile library for assessment.

3. An 'Independent sequence dataset' was generated by removing seed sequences from the 'Full sequence dataset' of the Pfam family. Individual sequences from the Independent sequence dataset were used as query and searched against profiles uploaded to FASSM in Step 2. Query sequences are annotated to a particular seed-sequence profile along with FASSM probability score.

4. Repeated the searches for all members in 'Independent Sequence dataset' to identify BRP. That seed-sequence profile which annotates a particular independent sequence query with high probability score is consider as the representative for the particular independent sequence.

5. Seed-sequence profile representative of all the independent sequences in a particular Pfam family were collected and seed-sequence profile that represents most independent sequences is considered as BRP for the family.

6. In a given Pfam family, if a single BRP derived from seed sequence data set does not have >= 50% coverage, the full sequence data set was considered for generating profiles. Further, a BRP was obtained from the profiles of Full sequence data using coverage analysis as shown in Figure 3.

7. For a given Pfam family, a single BRP was uploaded to the database.

### Coverage analysis

Coverage of an individual PSSM profile was calculated from the ratio between the numbers of independent sequence it annotates to the total number of independent sequences in the family. The coverage analysis formula is given in Figure 3. That PSSM which is shown to have the highest coverage, in identifying independent sequences, using the above method is considered as the BRP of the family. A single profile with the coverage >= 50% was retained as BRP of a given family. In families where BRP does not have a coverage value above the threshold value of 50%, the PSSM which has the highest coverage value in the family above the threshold is considered as the BRP for the family. In the case of protein families with two profiles, when both the profiles have the same coverage score, any one of the two profiles is selected and stored in the database. In a typical FASSM [9] run, motifs are identified in a query sequence in comparison to a set of family profiles. The motif segments are allowed to propagate to maximize the amino acid conservation scores. Further scores assigned for compatibility of the query sequence to the family profiles are for the presence of motifs, order and inter-motif spacing. FASSM performs the family association of a query sequence using the probability score of the family profiles.

Steps in Coverage analysis:

1. To retain BRP as representative for the family, it should have a coverage value above the threshold limit of 50.

2. In some families, BRP fails to cross the threshold limit; for such examples, the PSSM profile with the highest coverage value was considered as the BRP.

3. In some families, none of the PSSM profiles have coverage value above the threshold; in such instances, we have used only the domain of interest and generated independent sequences and re-examined the coverage.

4. In other instances, we have generated PSSM profiles from all the sequences available in the family (full set) and used all the sequences (domain length) as query for FASSM run.

5. Coverage was calculated for all the profiles, BRP was selected based on the highest coverage value.

### Best Representative PSSM profile (BRP) of protein families

**Figure 4:**
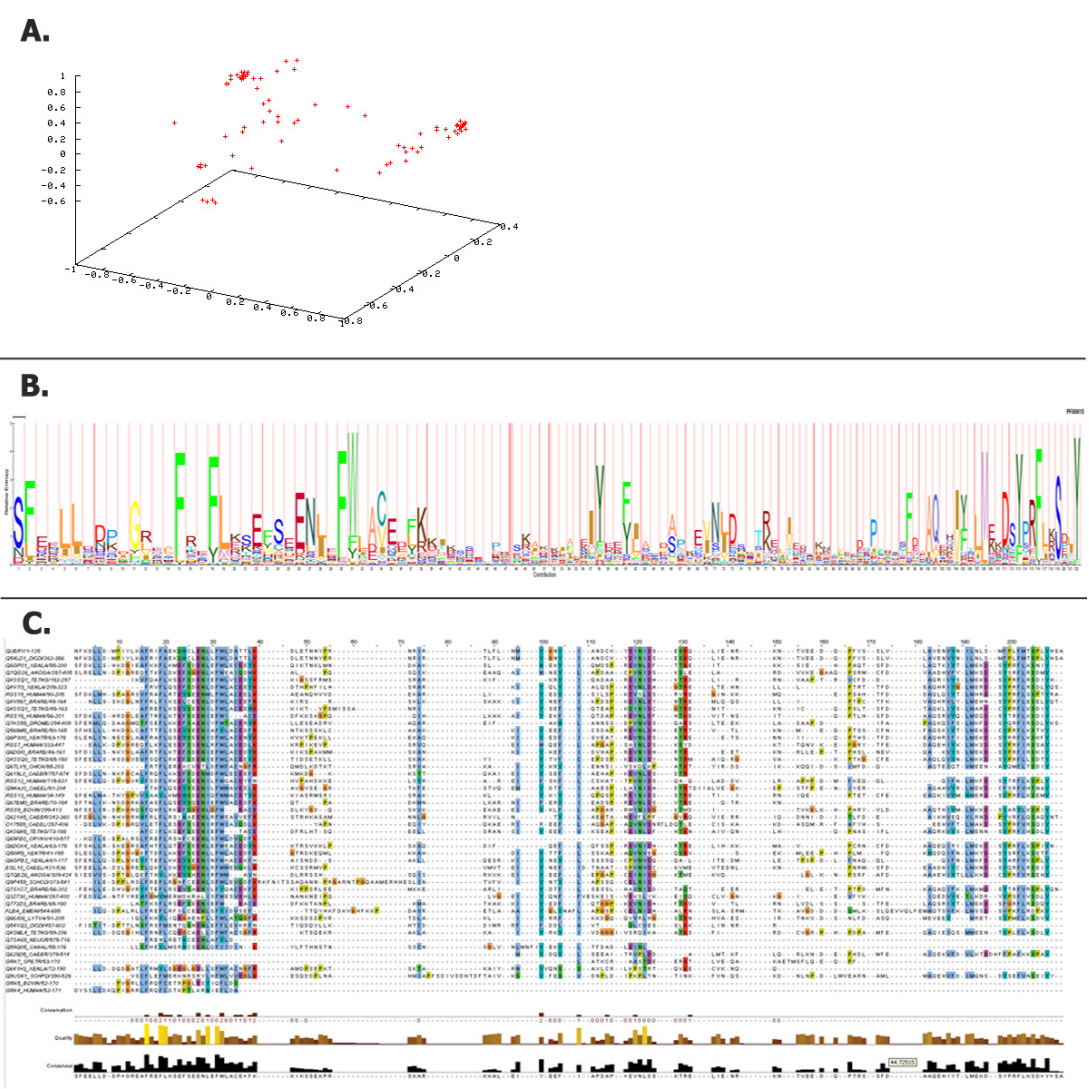
**PCA plot, HMM logo and alignment of RGS family (PF00615) generated using files from 3PFDB**.

We introduce a new concept called BRP in 3PFDB. 3PFDB is developed as a result of an attempt to generate single PSSM profile for any given protein families. BRP of a given family is generated by the curation and coverage analysis method explained earlier. BRP is generated from the reference sequence that encapsulates all the important information of a diverse or highly similar family to one single profile. BRP will be useful for researchers interested to perform large-scale protein family analysis. Protein family is a convenient level of sequence and structure based organization at which a group of proteins can be grouped to a family based on different features like domain, sequence conservation, functional motifs, and structural similarity. Each member of a protein family will agree with similar features, still a protein family can have a wide-variety of members ranging from highly similar to highly diverse members. Out of the 8524 (91.5% of Pfam version 22) BRPs reported in the current version of 3PFDB, BRPs are derived either from seed (7064, 82.9%) or full (1460, 17.1%) datasets. In case of entries which are derived from seed dataset, two types of profiles are mentioned in the database. For example in case of the example PF00001, two scores are provided in the database. Profile based on "Coverage of Best Profile" this refers to a profile that annotates seed queries with highest FASSM score in the results for a family. Profile based on "Best Profile Based on Coverage Score" refers to the profile, which is not the profile with highest FASSM probability score to annotate a query to a profile, but this profile annotates a larger set of seed sequence to the profiles of the family. But the profile based on "Coverage of Best Profile" is provided as the BRP of the family. For example, Acyl-CoA dehydrogenase, C-terminal domain [13-15] (Pfam ID: PF08028) 'Best Profile' is given as Q73YD4_MYCPA_236_369, this is the profile that annotates 83.19% of seed sequences with highest score, is ranked number one and hence selected as the BRP of the family. Q8XT96_RALSO_240_373 is given as "Best Profile Based on Coverage Score": this profile may not annotate a large number of seed sequences with higher probability (rank number one), but annotates a large number of seed queries in this case 91.70% (above threshold FASSM score). FASSM probability scores are derived from the conserved motifs detected from the profile. Motifs are identified in a query sequence in comparison to a family profile. Scores assigned for compatibility of the query sequence to the profile is based on the presence of motifs, order and inter-motif spacing. Further, FASSM uses the neural network routine to assign the final score for each profile. Detailed description about the scoring scheme and derivation of FASSM probability score is explained in an earlier work [9]. To illustrate an example of curation steps used in 3PFDB, we have provided the example of RGS family in Figure 1. PCA plot, hmm logo and alignment to describe the diversity of Regulators of G-protein Signalling family [16] (RGS family, Pfam ID: PF00615) is given in Figure 4. RGS proteins are multi-functional proteins with a major role in signal transduction [17]. The plot is generated using normalised alignment score from MALIGN [18] using GNUPLOT [19]. The alignment is curated from 'seed' alignment from Pfam and full length sequence is used for the generation of alignment. The plot clearly depicts the diversity within a protein family.

### 3PFDB database: excluded dataset

In the current version of 3PFDB, 9318 Pfam families were analysed and best-representative profiles were identified for 8,524 families. The remaining 794 families were excluded from the database due to its poor performance in the data curation steps. On further analysis of this excluded dataset, we have observed that due to the large number of sequences in independent sequence, seed-based PSSM profile was not able to annotate all the sequences in the given family and the average family coverage have been fallen below 50%. As we set 50% as the cut-off for the family coverage, this family will not be included in the database. Another reason for the exclusion is that the individual PSSM profiles of the family are not having 50% coverage value. In this scenario, the profile of a given family is unable to annotate half of the family members. In some of the excluded cases, like YonK protein [20] (Pfam ID: PF09642), Bacteriophage T4 beta-glucosyltransferase [21] (Pfam ID: PF09198) and *Mycoplasma arthritidis*-derived mitogen [22] (Pfam ID: PF09245), BRPs are not provided in the current version of 3PFDB. This is due to the limited number of sequences in these families and there is no suitable seed or member from the independent dataset to serve as BRP. Separately, if the family members are found to be identical, in such cases, any sequence from the family could be BRP and we did not provide BRPs for such examples in the current version of 3PFDB. List of protein families available in the current version of 3PFDB [23] and list of excluded families [24] are also provided in the database for easy access of the datasets. The current version of 3PFDB is corresponding to Pfam version 22 and 3PFDB will be updated periodically in response to the availability of newer versions of Pfam. A short delay in setting up the new version of the 3PFDB is anticipated due to the computationally intensive protocol used in the data curation steps. If users would like to perform BRP for custom generated alignments, users can contact the corresponding author for the FASSM program and other scripts used for the data curation.

### Database Design

**Figure 5:**
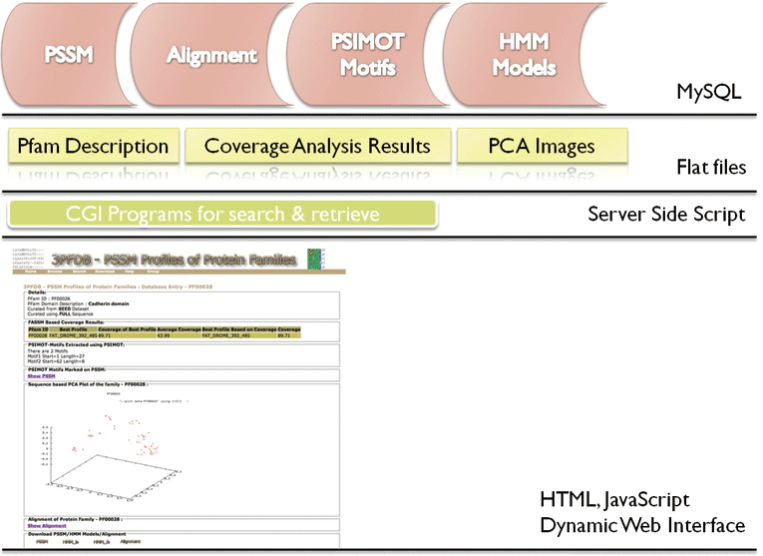
**3PFDB - Schematic representation of database architecture**.

3PFDB is developed on a MySQL [25] backend. Server side CGI scripts are coded in Perl [26]. Web interface is developed using HTML, and JavaScript. FASSM scripts are coded using a combination of C++ and Perl. ANNIE [27] version 0.5 neural network package was used to build neural network architecture. Blast version 2.2.16 [28] is used for PSI-BLAST [5] run and generation of PSSM profiles. BLAST generated alignments are converted in to PIR format using custom-Perl script. HMMER [29,30] version 2.3.2 - is used to create the hmm models. The normalised alignment scores to generate PCA plots are obtained using 'pca' routine from MALIGN version 4.0 [18]. Normalised alignment scores are used to generate the PCA plots using GNUPLOT 4.2 [19]. A schematic representation of the database architecture of 3PFDB is provided in Figure 5.

### Computing Time

The exploratory-search to identify BRPs for 9318 Pfam families were performed on a 32-node cluster powered by Athlon 64-bit Quad-core processors running on a CentOS operating system version 4. 10. 85,588 rigorous PSI-BLAST searches were performed on the cluster to identify the best-representative PSSMs for 8,524 Pfam families. To perform the PSI-BLAST search, 5 months of CPU hours utilised to perform the primary data curation step in the development of 3PFDB. 8,524 'hmmbuild' runs also were performed to generate hmm for the qualified family members.

### Database Content

For each entry in the 3PFDB, following information is provided.

• Details about Pfam family: 3PFDB also provides short description about the Pfam families included in the current version of the database. These details includes Pfam ID, Pfam Domain Description, Curated from SEED/FULL Dataset, Curated using DOMAIN/FULL Sequence, Number of Sequence in Seed Dataset, Number of Sequence in Full Dataset, Pairwise Identity of Sequence in Seed Dataset, Pairwise Identity of Sequence in Full Dataset, Number of reference sequence used to generate final profile

• FASSM based coverage analysis results: For every Pfam family members included in 3PFDB, the results from the FASSM runs are provided.

• PSIMOT-Motifs extracted using PSIMOT routine of FASSM: PSIMOT-Motifs refer to the conserved motifs obtained from a given BRP using the PSIMOT routine of FASSM.

• PSIMOT Motifs marked on PSSM: PSIMOT-Motifs predicted using PSIMOT routine of FASSM is highlighted on to the BRP of the protein family.

• Sequence based PCA plot of the protein family: PCA plot derived from the normalised alignment score from MALIGN is provided for each family

• Alignment of protein family in PIR format: Multiple sequence alignment based on the BRP in PIR format

• Download PSSM, HMM model and alignment: Users can download the PSSM(single BRP per family), Sequence alignment (in PIR format) from which the BRP is derived and HMM model (using the alignment from which the BRP is derived)

• Search options using Pfam family name, description and Pfam2GO annotations

### Search Utility

3PFDB offers two text based search options to search and retrieve PSSM profiles using different set of key words. The searches are designed using Pfam description and Gene Ontology [31] annotations derived from Pfam2GO [32]. Pfam2GO [32] is a useful way to map Pfam entries to GO. User can query 3PFDB using Pfam ID, Pfam description, Pfam short-description and Gene Ontology [31] related terms like GO ID or description. User can search the database using key words related to Pfam description and Pfam2GO annotation and retrieve all the profiles that related to the key. As family specific and function-specific analysis is gaining importance in bioinformatics, availability of search engines to query 3PFDB using Pfam description and Gene Ontology will be useful.

## Discussion

Several tools and databases employ PSSMs for different application in bioinformatics. These include but not limited to homology searches, pattern search, function assignment, function annotation, transcription factor binding site prediction, protein family classification using machine learning approaches like support vector machine. PSSMs are employed in several bioinformatics studies in different applications [33-36], for example predicting cyclin protein sequences [37], predictions of human, mouse and monkey MHC class I affinities for peptides [38], prediction method for virulent proteins in bacterial pathogens [39], sequence alignment and fold recognition with a custom scoring function [40], sequence-based prediction of DNA-binding residues in DNA-binding proteins [41], prediction of sub-cellular localization of gram-negative bacteria proteins [42]. Bioinformatics tools and databases like PROSITE [43], PRINT [44], BLOCKS [45] etc. employ PSSMs for pattern recognition based applications. CDD [46] provides a collection of PSSM profiles for Pfam families and MulPSSM [47] is another related resource that use multiple PSSMs corresponding to a given alignment and variable reference sequences. Databases like MULPSSM [39] have demonstrated the effectiveness of searching exhaustively, from different starting points and query sequences, in order to improve coverage. However, the total number of protein sequence domain families in databases like PFAM is far too high to handle all individual sequences. Owing to distant relationships and huge sequence dispersion within protein families, it is not always easy to find representative sequences. The concept of 'seed' sequences within Pfam databases is useful but does not, for many protein families, assure high and uniform coverage. The Pfam database [12,48] is a large collection of protein domain families, each represented by multiple sequence alignmentsand hidden Markov models (HMMs). Pfam database is divided in to two levels depending up on the quality of the families as Pfam-A and Pfam-B. Pfam-A is derived from the UniprotKB [49] derived sequence database 'Pfamseq'. Each Pfam-A family consists of a curated 'seed alignment' containing a small set of representative members of the family, profile hidden Markov models (profile HMMs) built from the seed alignment, and an automatically generated full alignment, which contains all detectable protein sequences belonging to the family, as defined by profile HMM searches of primary sequence databases. Pfam-B families are un-annotated and lower quality automated alignments generated automatically from the non-redundant clusters of ADDA [50]. In the current version of 3PFDB [11], we have used the seed alignment as the primary dataset to identify the BRP of a given protein family. Sequences from 'seed dataset' are used as reference sequences to identify BRPs for 7064 families. As this covers only 75% of Pfam version 22, we further used the sequences from 'full dataset' to identify BRPs of 1460 families (16%). As we assessed individual profile by its efficiency to annotate more than 50% of sequences in independent dataset in the case of 'seed dataset' and all sequences within a family in case of 'full dataset', the BRPs are one of the most authentic representation of a family in a profile format. This important information about the source ('seed dataset' or 'full dataset' and the type of sequence ('domain' or 'full') from which the BRPs are derived is provided in the 'Details' section of entries in 3PFDB. As the selection of a single, best representative PSSM from different members of the protein sequence families are currently performed in a non-standardized way, the data mining approach used in this work is a primary attempt in this regard. The data mining approach used for the selection of BRP is novel, yet a generic method which and can be employed in general, with any program of choice that uses PSSMs.

3PFDB provides a single best seed representative for the 91.4% of protein families reported in Pfam database (version 22) and thereby immensely reduces the computational time for sequence searches and to establish relationships to the ever-expanding databases of sequence domain families. Further, the choice of the best seed representative using FASSM ensures best coverage since none of the seed sequences may uniformly attain high coverage. 3PFDB offers coverage analysis results for the Pfam family with other features of the database. For example, the coverage analysis results of the RGS family (Pfam ID: PF00615) [51] clearly indicates that the BRP using the sequence is derived from 'Q54LD1_DICDI_262_386', this sequence was able to annotate 377 reference sequence with a coverage of 76.78%. Average coverage of this family starting from the seed sequences, however, was only 44.42%.

## Conclusion

A new data mining approach to identify a single BRP for protein families available in Pfam database is designed. The data mining approach is applied to Pfam version 22 and a new database of Best-Representative PSSM profiles (BRPs) of protein families called 3PFDB is developed. To the best of our knowledge, 3PFDB is first of its kind resource of BRPs generated using PSI-BLAST [5] and assessed through coverage analysis results of the sensitive sequence based annotation method FASSM [9]. PSSMs, alignments and HMM models available from 3PFDB can be extensively used for studies that require family-specific PSSM profiles.

## List of abbreviations used

3PFDB: Database of best-representative; PSSM: Profiles of Protein families; BRP: Best Representative; PSSM: profile of a protein family; FASSM: Function Association using Sequence and Structural Motifs; HMM: Hidden Markov Model; PSI-BLAST: Position Specific Iterative - BLAST.

## Competing interests

The authors declare that they have no competing interests.

## Authors' contributions

RS conceived of the study and discussed the approaches. KS developed and organised the database. KS and PN had written the scripts and performed the calculations. KG had developed the FASSM algorithm; Both KS and PN wrote the first draft of the manuscript. KG and RS provided critical comments to the manuscript. All authors read and approved the final version of the manuscript.
